# Chinese Tertiary-Level English as a Foreign Language Teachers’ Emotional Experience and Expression in Relation to Teacher-Student Interaction

**DOI:** 10.3389/fpsyg.2021.759243

**Published:** 2021-10-27

**Authors:** Xinfeng Xie, Guiying Jiang

**Affiliations:** ^1^College of Foreign Languages and Cultures, Xiamen University, Xiamen, China; ^2^School of International Education, Guizhou Normal University, Guiyang, China

**Keywords:** Chinese tertiary-level EFL teachers, emotional experience, emotional expression, teacher-student interaction, positive psychology intervention

## Abstract

The present study examines the emotional experience and expression of Chinese tertiary-level English as a foreign language (EFL) teachers and their interaction with their students. Data were drawn from semi-structured in-depth interviews with 10 EFL teachers recruited from seven universities of different levels in China and were analyzed in light of Emotional Geography Theory. The results reveal that Chinese tertiary-level EFL teachers experience more negative emotions than positive ones. The emotions most frequently reported by them are anger, enjoyment, anxiety, disappointment, and ambivalence. When it comes to emotional expressions, Chinese tertiary-level EFL teachers tend to display positive emotions by following the emotional rules of school settings. This study also uncovers that EFL teaching in Chinese universities is characterized by EFL teachers’ physical and moral distance from but political closeness to students, all of which are the sources of EFL teachers’ negative emotions. The need for providing positive psychology intervention for EFL teachers is then suggested.

## Introduction

English as a foreign language (EFL) teachers lead a diverse emotional life in school settings. In applied linguistics and TESOL, extensive studies on EFL teacher emotions have been documented in the literature (e.g., Cowie, 2011; Hiver and Dönyer, 2017; Martínez Agudo, 2018; Miller and Gkonou, 2018; Dewaele and MacIntyre, 2019; Gkonou et al., 2020; Dewaele and Li, 2021), revealing the non-cognitive nature of FLT as well as the psychological self of EFL teachers. Recent studies have shown that positive language teacher emotions, such as enjoyment, care, and love, can be pedagogical strategies for improving second/foreign language learning and teaching (Derakhshan et al., 2021; Wang et al., 2021). In terms of the dynamicity in EFL classrooms, teacher emotions are regarded as a force of creating classroom climate which affects students’ interest in and motivation for learning (Toraby and Modarresi, 2018). Dewaele and Li (2020) confirm that emotions guide and shape teacher-student interaction by transmitting between them. In this regard, EFL teacher emotions have an impact on the interaction between teachers and students. Reversely, the understanding and misunderstanding incurred from the interaction between group members will give rise to various positive and negative emotions (Liu, 2016).

Seeing the interrelationship between EFL teacher emotions, teacher-student interaction, and EFL teachers’ instructional strategies, the study aims to depict the emotions experienced and expressed by Chinese tertiary-level EFL teachers while interacting with students, and to excavate the antecedents of their emotional experiences in light of Hargreaves’ (2000) emotional geography theory. Similar studies have been done by Xu (2013) and Liu (2016), but the present study is different from theirs in that their studies focus on emotions experienced by EFL teachers while interacting with students, colleagues, and parents, etc. Yet the present study focuses on EFL teacher emotions experienced and expressed while interacting with students in the classroom. Another difference lies in the fact that the participants in Xu’s study are English teachers recruited from high schools in southern China and the only participant in Liu’s study is an immigrant ESL teacher in England. Therefore, the present study is the first article that depicts teachers’ emotional lives in Chinese tertiary-level EFL classrooms. We mainly address the following four research questions:

1. What emotions are experienced by Chinese tertiary-level EFL teachers while interacting with students in the classroom?
2. What are the antecedents of emotions experienced by Chinese tertiary-level EFL teachers?
3. How do Chinese tertiary-level EFL teachers express their emotions?
4. What are the patterns of teacher-student interaction in Chinese tertiary-level EFL classroom?

## EFL Teacher Emotion

Enjoyment, pride, anger, anxiety, shame, boredom, and pity are the most salient teacher emotions reported in school settings (Frenzel, 2014). However, EFL teachers’ emotional experiences are different from those of teachers teaching other subjects. Horwitz (1996) discovers that “non-native” English speaking teachers experience peculiar anxieties because of their language deficiencies. Along the same vein, EFL teachers’ emotions, especially anxiety, are acute and prominent because the language they teach is not their or their students’ mother tongue (e.g., Ma, 2012). All these studies point to the fact that EFL teacher emotions are subject-specific.

It must be noted that not all emotions experienced by EFL teachers are negative. For example, EFL teachers feel happy with applying new technologies into EFL teaching (Azzaro and Martínez Agudo, 2018), and frustrated and exhausted as well as satisfied in the process of assessment (Brown et al., 2018). Yan and Tan (2018) also report that French teachers of Chinese experience diverse, complex, and changeable emotions, including love, joy, sadness, anger, fear, and so on, in their professional lives. What is more, EFL classroom is full of emotion affordances. In other words, different teaching environments cause diverse emotional experiences of teachers, which entails that teacher emotions are context-specific in EFL learning and teaching. For example, previous studies have addressed EFL teacher emotions in relation to decision making in the selection and adaptation of ELT materials (Tomlinson, 2018), the evaluation process of special education (Dunn and Ernst-Slavit, 2018), and ESL teachers’ interaction with the school community (Nguyen, 2018). Among the various sources of EFL teacher emotions, interaction with students in EFL classroom is probably the most important one. Xu (2013) suggests that the emotional rewards of teaching are mostly from students. Alzaanin (2021) echoes that relationships in the classroom influence the construction, expression, and communication of teachers’ emotions. In a word, these studies cover a range of emotional constructs, such as positive and negative emotions, emotional labor, burnout, and emotional intelligence, suggesting the context-specificity, multidimensionality, and complexity of EFL teacher emotions experienced in diverse contexts of EFL teaching.

In contrast, not much research in applied linguistics has been done on EFL teachers’ emotional expression. Among the few studies, EFL teachers’ emotional expression is usually discussed in terms of emotion regulation. Hypothesized as a key determinant of effective performance (Cicchetti et al., 1995), emotion regulation is such a behavior that changes the quality, intensity and length of emotion, the time to experience, or the way to express (Gross, 2015). One of the most popular theories of concern on emotional expression is the process model of emotion regulation, proposed by Gross (1998), who identifies five kinds of strategies for regulating emotions: (a) situation selection, (b) situation modification, (c) attentional deployment, (d) cognitive change, and (e) response modulation. Based on this model, Morris and King (2018) suggest that cognitive reappraisal reduces frustrations experienced by EFL teachers at a university in Japan. Similarly, Morris and King (2020) posit that emotions are regulated contextually. For example, teachers adopt situational strategies to maintain positive emotions, attention deployment strategies to afford emotional gratification, cognitive reappraisal to adapt to classroom stressors, and response modulation to support teaching goals and relationships. Talbot and Mercer (2018) discuss the emotional regulation strategies that university EFL teachers in the United States, Japan, and Austria adopt to manage their emotional wellbeing. In their terms, the regulatory strategies they use are social in nature, as a result of which they propose employing social comparisons in helping teachers focus on the positive aspects of social positioning. These studies show the possibility of conducting positive psychology intervention in regulating EFL teacher emotion and promoting their wellbeing.

Besides, EFL teachers’ emotional expression is more often discussed in terms of emotional labor. Emotional labor refers to the conflict between feeling rules prescribed by an institution and its employees’ internal feelings (Hochschild, 1983). Its central tenet is “a dichotomy between real and manufactured feelings as well as a split between internal feelings and external expression of them” (Benesch, 2017, p. 40). In this sense, emotions are not viewed as individual inner states but rather as feelings experienced and performed according to the feeling rules of particular situations. Though the feeling rules of schools and classrooms are usually implicit and recognized subconsciously, they shape what EFL teachers believe they should feel and how they should display emotions. For example, EFL teachers have to suppress negative emotions when faced with a low level of student attention in class but exaggerate positive emotions to encourage students to practice speaking in English (Kim and Kim, 2018), even though their real emotions might be disappointment and anger. Similarly, while interacting with students, parents, and institution, EFL teachers conduct emotional labors by displaying positive emotions out of the intention of upholding their professional role as a teacher (Cowie, 2011).

In short, literature is predominated by studies on EFL teachers’ emotional experiences but dotted by studies on EFL teachers’ emotional expressions. Another fact is that few studies tend to include EFL teachers’ emotional experience and emotional expression in a single study. The current study puts them together out of the following considerations: (1) emotional experience and emotional expression are the two constituting parts of EFL teachers’ emotional lives. A lack of either one would make the depiction of their emotional lives incomplete; (2) the inconsistence between EFL teachers’ experience and expression of emotions will lead to their emotional labor (Zhang and Zhu, 2008), which will in turn cause new experience of emotions, negative ones in particular; and (3) emotional expression is actually the most important guide for the external recognition of an individual’s emotions. Teachers’ emotional expression has an impact on what or how individuals perceive what a good “teacher” is (Karl, 2009). It might be true that EFL teachers’ emotional experiences are a factor indicating their emotional wellbeing, but considering the significant influence of EFL teachers’ emotional expression on the effectiveness of EFL teaching, it might deserve more of our effort to investigate EFL teachers’ emotional expression.

## Emotional Geography Theory

Positive emotional climate in the classroom depends on mutual emotional understanding between teachers and students. Teachers usually listen to and scan their students, checking students’ verbal and nonverbal expression of emotions in order to respond to and engage students by way of emotional understanding, a symbol of teacher quality. Hargreaves (2000) proposes emotional geography theory to examine the emotional understanding between teachers, students, colleagues, and parents. The spatial and experiential patterns of closeness and/or distance in their interactions “help create, configure and color the feelings and emotions we experience” (Hargreaves, 2000, p. 7).

Aiming to identify the supports for and threats to the emotional understanding of schooling, Hargreaves (2001) identifies five sub-groups of emotional geographies: physical, moral, sociocultural, professional, and political. Physical geography refers to the closeness and/or distance created by time and space. Physical distance occurs when teachers spend little time in interacting with students. Moral geography refers to the closeness and/or distance configurated by different purposes in teaching. If teachers find their purposes different from their students’, moral distance is configurated. Sociocultural geography refers to the closeness and/or distance created by background differences, such as gender, age, ethnicity, and culture. Sociocultural distance takes place when these differences cause disconnection between teachers and students (Cil and Dotger, 2017). Professional geography refers to the closeness and/or distance created by different understandings of the norms of professionalism. The traditional masculine model of professionalism creates a distance between teachers and students since it stresses that a distance must be kept to indicate authority and professionalism. Political geography refers to the closeness and/or distance created by different understandings of power. In classroom, teachers who wield too much of their power will withdraw themselves away from students and give rise to emotions, such as indifference, stress, and frustration.

In consequence, emotional geography theory is based on the distance and/or closeness configurated in the process of teacher-student interaction. The distance and/or closeness of a certain geography will evoke particular emotions. For example, teachers experience happiness with students’ parents when they received parents’ gratitude, appreciation, agreement, and support – indicators of moral closeness between teachers and parents (Hargreaves, 2001). In this sense, it can be used to examine EFL teachers’ emotional lives when they are interacting with students in the classroom.

## The Present Study

A bulk of approaches have been employed to study teacher emotions, among which are qualitative ones like interview, case study, narrative inquiry, and quantitative studies like questionnaire survey. Qualitative approach, such as interview, is praised for providing insights of a phenomenon, which would facilitate more in-depth understanding of the complexity of human experience. The interview-based approach is also effective in probing into the emotional lives of EFL teachers and providing the reasons for the experience of certain emotions. As a result, the qualitative approach is adopted in this study with the guidance of emotional geography theory (Hargreaves, 2000) so as to keep consistency with his study and make the results comparable.

### Participants

This study followed the “Principle of Purposeful Sampling” (Patton, 1990, p. 169) and selected 10 tertiary-level English teachers for non-English major students as our participants (see Table 1). These in-service teachers (five male and five female) were chosen from seven Chinese universities at local, provincial, and national levels. The selection of these teachers followed the principle of “maximum variation” (Creswell, 1998), and full consideration had been given to their differences in age, gender, educational background, professional title, years of teaching experience, etc. Among the 10 teachers, two of them were novices in teaching English at university with an experience of less than 1year and the others’ teaching experiences ranged from 5years to 11years. Two of them had received doctoral degrees and the rest were Ph.D. candidates. As for their professional title, there was one assistant professor, one associate professor, and eight lecturers.

**Table 1:** Demographics of participants.

Subjects	Gender	Professional titles	Years of teaching experience	School types
T1	Female	Lecturer	Less than 1year	Provincial
T2	Male	Lecturer	5years	Provincial
T3	Female	Lecturer	6years	Local
T4	Female	Lecturer	8years	Local
T5	Female	Lecturer	7years	Local
T6	Male	Lecturer	6years	Provincial
T7	Male	Lecturer	11years	Provincial
T8	Female	Associate professor	9years	Provincial
T9	Male	Assistant professor	1year	National
T10	Male	Lecturer	7years	Provincial

### Data Collection and Analysis

Ten EFL teachers were interviewed with a time range of 45–70min about their interaction with students in and after class, about their emotional experiences during the teacher-student interaction, about the causes of their emotions, and about their ways of expressing and dealing with the emotions. Questions like “How do you feel about that?” are strictly excluded since it is confusing for the interviewees whether to give an answer about opinions or feelings, especially in this study. Instead, “What is your opinion about that?” or “What do you think about it?” is used to ask about their opinions, while “What emotion did you feel or experience at that moment?” is used to ask about their emotions (Patton, 2002). The interviews were conducted in Chinese and recorded with the teachers’ consents.

Extracts were first selected and categorized according to five themes that have been identified as components of emotional geographies by Hargreaves (2000): physical geography, moral geography, sociocultural geography, professional geography, and political geography. We coded and reconstructed the data under these themes. For instance, “classroom questioning” was extracted from “I was going through some emotions when I asked questions and the whole class kept silent” and classified into physical geography; while “generation gap” was extracted from “…students prefer to talk to peers rather than teachers…what they like is different from what we (teachers) like…” and grouped into sociocultural geography. In the process, several subthemes were generated between the five major themes and the excerpts. Under sociocultural geography, for example, we identified such subthemes as “generation gap” and “family condition.” Data analysis was carried out with NVivo 12.0.

Triangulation was employed to ensure the reliability and validity of the data. All 10 interviews were transcribed verbatim in the language they were conducted in and were double-checked by an expert in EFL teaching so as to eliminate mistakes. Each text was submitted to the corresponding teacher participant for verification. Furthermore, the transcribed data were intercoded by the two authors and intra-coded by one author with an interval of 2months. Specifically, to achieve intercoder reliability, the two coders got together after the first round of independent coding to discuss their results with an open attitude. At this stage, codes made by independent coders were discussed and those that were the same were kept and those different were negotiated to generate more suitable codes that would lend themselves to analysis. One coder recoded all the transcripts 2months later so as to check for intra-coder reliability. In the first round, the intercoder reliability coefficient was 0.89, and the intra-coder reliability was 0.91. After the coding scheme was refined, both reliability coefficients were improved and reached a level with higher reliability. Inter-coding and intra-coding were adopted because they are particularly useful in content analysis of open-ended survey responses like interview transcripts since without intercoder and intra-coder reliability, the interpretation of the content cannot be considered objective and valid (van den Hoonaard, 2008).

## Results

### EFL Teachers’ Emotional Experiences

The emotions are presented in Table 2 in terms of their frequency mentioned by the 10 teacher participants. As is shown in the table, these teachers experienced more negative emotions than positive ones. Negative emotions most frequently mentioned by them are anger, anxiety, disappointment, ambivalence, and helplessness, while positive emotions are enjoyment and pride. The result shows, to a certain degree, that EFL teachers in Chinese universities are dominated by negative emotions in the in- and after-class interactions with students, which might be one of the factors causing EFL teacher burnout. Besides, these teachers also reported emotions, such as indifference, contempt, disgust, pity, and awkwardness, each of which was mentioned only once, which are excluded from the present study.

**Table 2:** Discrete emotions experienced by Chinese EFL teachers.

Emotions	Anger	Enjoyment	Anxiety	Disappointment	Ambivalence	Helplessness	Pride*
Frequency	70%	80%	40%	40%	30%	20%	20%

*Pride is asterisked for its controversy with enjoyment. (Teacher participants regarded the same emotional incidents as either pride or enjoyment.)*

It is also shown that similar to Frenzel et al.’s (2016) study, anger, enjoyment, and anxiety are listed as the top three most frequently experienced emotions by teachers. Meanwhile, disappointment and ambivalence are seldom recorded in the existing literature, making them unique to Chinese EFL teachers. Emotions included in Table 2 will be discussed more specifically in relation to the situations where these emotions may occur.

#### Positive Emotions

The positive emotions experienced by the teacher participants are enjoyment and pride. Eight out of 10 teachers reported that they had experienced enjoyment while there were only two in 10 who had experienced pride.

##### Enjoyment

Enjoyment is one of the most salient positive emotions (Sutton and Wheatley, 2003; Frenzel, 2014) experienced by teachers in teaching context. Being positive, or specifically being joyful, is regarded as one of the basic requirements of being a teacher in China. In addition, our investigation also shows that some teachers were aware of the importance of enjoyment in the classroom. T5, for example, believed that enjoyment was a driving force for students’ initiation.

*T5*: Happiness was perhaps a positive emotion demonstrated in the classroom. … to make the class nicer, not to spoil students’ initiative. In addition, I think nowadays students are more sensitive to this emotion.

Enjoyment usually comes from students’ good performance in the classroom. Frenzel (2014) suggests that the antecedent of teacher emotions is their expectation of students’ behavior. Teachers come to the classroom with expectations of students’ behaviors as shown in the example of T2.

*T2*: Sometimes I was happy. For example, some students were brave enough to express their opinion, which made me very happy. … After asking a question, his/her answer … met my expectation, that is, met the teaching effects that I expected. …

*T9*: … Sometimes it was rather dull in the classroom. The silence made me unhappy. There were happy moments too. It made me happy when they participated in discussion, especially when the discussion went well and met my expectation.

*T10*: If his/her answer was good enough, … if he/she had to answer the open-ended questions, he/she might have his/her own answers different from the textbook. I was happy to hear these answers. … I was also happy if students could complete the assignment earnestly.

The above excerpts show that students’ unexpected academic performances which were beyond teachers’ expectation increased teacher enjoyment. It also reduced teachers’ negative emotions and protected them from being emotionally exhausted (Taxer et al., 2019). Unfortunately, this cross-sectional study could not reveal the dynamism of teacher enjoyment. An illuminating longitudinal study conducted by Frenzel et al. (2018) shows that teacher enjoyment changes with the going on of teaching in a school year. For example, at the beginning of the school year, teacher enjoyment was positively related to students’ perceptions of teachers’ enthusiasm, while at midterm, it was positively related to student enjoyment.

*T3*: I felt happy because what I said or did has influenced him. … One of my former students called me the other day, saying that she was going to graduate from the university and wanted to thank me for I was the one who had influenced her most in the past four years.

*T4*: The students whom I instructed participated in all kinds of competitions and won prizes. This was what made me happy.

Students’ evaluation on teachers and their achievements are also sources of teacher enjoyment. T3 and T4 reported that their students’ appreciations or achievements were the source of their enjoyment. Chinese EFL teachers often instruct students to participate in speaking or writing competitions. Honor and rewards for teachers usually come with students’ achievements. Chinese tertiary-level EFL teachers will be evaluated at the end of a semester, and the result of students’ performance in competitions will be considered as one of the criteria to measure teachers’ performance, which in turn affects their bonuses and honors of the school year. In consequence, the higher standard of the competition is the happier the teachers will be.

*T1*: … I was happy with or appreciated their attitude. For example, there was a student who always sat in the first row while the others were all crowded at the back of the classroom. …

Another source is from students’ attitude toward the subject as well as their teachers as indicated by T1. Unlike teachers at primary- or secondary-level, students’ grade is not the upmost concern for Chinese tertiary-level EFL teachers. What worries them more is the fulfillment of the teaching goals and their interaction with students. Therefore, a student who shows a positive attitude toward the teacher or the course, be he/she good at English or not, will make the teacher happy.

##### Pride

Pride is the second most relevant positive emotion for teachers in this study and is closely related to enjoyment. It is another positive emotion included in this study for further discussion not because of its high frequency mentioned by teacher participants but because of the ambiguity between enjoyment and pride.

*T7*: There is a saying which goes that everyone has his own brand. In my first few years as a class advisor, indeed, especially the first few years, my students had won the first prize of all kinds of competitions. I felt that my life was not wasted. … I was satisfied and proud.

*T8*: I was very proud when a student told me it was me who encouraged him constantly and helped him build confidence.

After checking all of the codes subsumed to pride, only the above two excerpts were found. It seems that students’ achievement was one of the few factors that contributed to EFL teachers’ pride. It has been discussed that T3 and T4 regarded students’ achievements as sources of enjoyment. But T7 and T8 deemed them as sources of pride, as shown in the above excerpts. It seems the same emotional incident may function as an antecedent of different emotions.

The difficulty to tease pride out from enjoyment shows the nuanced and overlapped components of teacher emotions. One possible explanation to this phenomenon could be that EFL teachers hold different levels of expectation. Some teachers regard a certain threshold value of expectation as corresponding to enjoyment, while others might regard the same value as related to pride. This may account for the much lower frequency of pride than that of enjoyment.

#### Negative Emotions

As is shown in Table 2, anger, anxiety, disappointment, and ambivalence are the top four negative emotions most frequently mentioned by teacher participants.

##### Anger

Anger is the most common negative emotion reported by teacher participants. Teachers in EFL classroom may fly into a rage because of many factors, among which are students’ indiscipline, challenges, unreasonable requests, and failure to meet teachers’ expectations. For example, teachers might be mad at their students for indiscipline or misbehavior in the classroom which is the major antecedent of anger (Frenzel, 2014).

*T10*: I experienced more emotions when I taught those who were majoring in physical education or arts. These art students, for example, were completely those who did everything by instinct. They would do what they wished. They would go out of the classroom or go to the restroom without asking for my permission. I would criticize them, but they seemed to have a memory as short as a fish. They completely ignored the criticism. I was very angry in the first year of my teaching career, but gradually the rage was fading.

*T3*: I encountered a boy student holding a girl student in his arms in the classroom. I fell into a rage immediately, questioning them how come that scene should happen in the classroom. I had a bad tone at that time, and then I even felt a sense of disgust.

T10 was from a third-tier university located in southwestern China. His report reflected at least part of the reality of Chinese EFL class at tertiary-level. To him, indiscipline might be the first and foremost reason that led to anger of Chinese tertiary-level EFL teachers. He was expecting university students to be mature enough to behave themselves in the classroom. The fact, however, was that some students tended to ignore the classroom rules by eating breakfast, chatting in twos and threes, or walking out of the classroom without making any excuse. These phenomena usually happened among students of certain majors like physical education, music, or fine arts. Or, in a broader sense, they came from universities or colleges of low level or in underdeveloped areas, such as the western, southwestern, or northwestern China. T3 who is from northwestern China also recalled a situation that made her particularly angry. She could not tolerate the intimacy appearing in the classroom. She was angry and even disgusted when seeing the intimate hugging between a boy and a girl student. The classroom is a serious, if not sacred, place for her, which cannot be disdained by improper behaviors.

*T4*: I was angry that some students turned a deaf ear to my words and argued with me that it was my arguments that were groundless. … It seemed as if he was always not on the same tune with me. It really pissed me off.

*T8*: …I would definitely be angry if my students dared to challenge my authority by questioning what I asked them to do.

Anger is also believed to be generated by student challenges. Some teacher participants like T4 and T8 regarded student challenges to their authority as offensive, which made them angry. But the emotions related to student challenges could be different according to what students challenge for. Student challenges could be those related to teacher power and discipline or to teachers’ knowledge. The former two would usually generate anger, while the latter would lead to anxiety or awkwardness.

*T7*: … I was angry that a university student should make mistakes in writing a name on the certificates of awards. … I got mad when I found that my students were sloppy in doing their homework or copied their classmates’ homework.

*T9*: In general, there was always one or two students who would take the initiative to ask questions. It was good. But in some classes, the climate would be dull. I was unhappy at the beginning, then I had to call on some students to answer the questions. Then it was getting better. …

*T10*: … sometimes I had to explain some abstract concepts. After explaining for over ten minutes, a student stood up asking what I had been talking about. Wasn’t it furious? …

Students’ failure to live up to teacher participants’ expectation is another factor that made them angry. Teachers usually assigned tasks equal to their students’ cognitive ability as well as according to their teaching goals. When these tasks, such as answering questions or writing names on the certificates of awards, could not be done properly, these teachers tended to be angry. They also felt angry when they realized that their efforts made no difference in students. After explaining an abstract concept to his students with great efforts, T10 was expecting to make himself understood by his students, but what made him angry was that the students were still in puzzle.

*T2*: Teachers had to follow certain criteria while scoring. It was impossible for me to lower the standard because of one or two students’ request. It was unfair to those who also failed in the exam. … I felt unhappy, if not angry, with it.

Although most Chinese tertiary-level EFL teachers make it clear that they will not change students’ score of the final examination, students still make requests for changing scores out of various reasons such as not wanting to get a low GPA, wanting to go abroad, or applying for the membership of Chinese Communist Party. Most teachers usually ignore such requests but will also be annoyed if their cell phones or e-mail boxes are bombarded constantly by students’ messages or mails. T2 reported his unhappy experience with this regard.

##### Anxiety

Anxiety has long been the most extensively studied emotion in applied linguistics since the 1990s (Horwitz, 1996). It is commonly acknowledged that teachers are anxious when they are incompetent enough in teaching (Darby, 2008), lesson planning, and classroom management (Chang, 2009). EFL teachers experience anxiety when they deliver a lesson in another language or to a group of EFL students, or because they are novices and in lack of experience in teaching and managing a class.

*T4*: … I was nervous when I was a novice teacher. …

*T6*: … At the beginning of my teaching, I felt nervous because of lack of experience. The poor interaction with students would also make me disappointed…. On entering the classroom, I was very nervous and excited as well. I was going to start a new journey. …

*T9*: It was my first formal class, before which I had only taken over several classes for a teacher on leave. I was a bit nervous in the first semester. … It was difficult to say what the reasons were. It was probably because I had to face so many students.

Student teachers and young teachers are more likely to fall victim to anxiety (Sutton and Wheatley, 2003; Chang, 2009). T4, T6, and T9 have reported anxiety experienced when they were novice teachers. T6 and T9 also expressed their anxiety about interacting with new students. T6 is a Ph.D. candidate majoring in English Language and Literature and T9 is a doctor majoring in Foreign Linguistics and Applied Linguistics and graduated from an Australian university, so it was not their language proficiency that made them anxious. Both of them admitted that they were introvert and afraid of interacting with strangers. This indicates that not all teacher emotions are related to teaching itself. Teacher anxiety is also concerned with teachers’ personality.

##### Disappointment

Similar to anger, teachers experience disappointment because of students’ failure to meet their expectations. If such feeling is experienced intensely, it might glide to anger. In this sense, expectation is the underlying factor driving EFL teachers to experience emotions from enjoyment to pride on the one end and from disappointment to anger on the other end.

*T2*: I was expecting students to interact with me and with their classmates. I wanted to see more communication between them. But they did nothing. I was really disappointed.

*T7*: To be honest, if the students couldn’t meet my expectation, I was indeed very disappointed. … It was because students’ language proficiency was really low. To me, teaching them is pain without gain.

Students’ failure to live up to teachers’ expectation was a prominent reason that led to disappointment among teacher participants. T2 was disappointed for not seeing students interact with him actively in the classroom. T7 even directly expressed his disappointment when he found that his devotion of time and efforts in teaching was not well paid off by students’ performance.

*T1*: I was always worried about them, thinking why they were late for class, what the matter was, and why they hadn’t come … I realized that they didn’t value English class much and that they were floppy. So, I was disappointed.

*T6*: I felt that students didn’t show their respect to me. I had prepared for the lessons and considered every teaching step, but the students didn’t give me any response. I was disappointed at them.

Students’ indifference in English itself, the course, and teachers’ work is another factor that leads to EFL teacher disappointment. T1 was worried about her students on a rainy day, thinking about different situations that had stopped her students from coming to the classroom. Yet, she could not hide her disappointment when she realized that her care and love for students were worthless since students thought the English class was not important and not worthy of their getting wet. Similarly, students’ disrespect toward the teacher and the course made T6 disappointed.

##### Ambivalence

These teacher participants were likely to find themselves in a dilemma while interacting with students. In China, teachers are required to be positive and show their students with love and care by laws, regulations as well as the ethics of teaching. They have to hide their negative emotions and pretend to be happy. However, teaching does not always give teachers positive psychical rewards. The phenomenon that teachers are struggling between true feelings and faked feelings is demonstrated in T6’s report.

*T6*: Teachers should be emotionally positive rather than negative in the classroom. This is my understanding of the teaching profession. I don’t think one is a qualified teacher if he/she wears anger or disappointment on the face. A teacher should stage positive emotions while hiding negative ones, which is a basic quality that a teacher should possess. … I know I have to act in this way, but it always makes me torn.

On the other hand, the scoring system in Chinese EFL teaching at tertiary-level is different from that at primary- or secondary-level. Students’ score of a course consists of two parts, namely, the score of daily performance accounting for 20–40% and the score of the final exam paper taking up 60–80%. This system aims to evaluate students dynamically in the learning process rather than with a single final score of an exam paper. It is also this system that gives teachers the right to change students’ scores, which may be a source of teacher emotions. Moreover, there are other specific scoring systems which are prescribed by a university. In the interview, T9 explained a scoring system carried out in his university.

*T9*: Score seems to be one of the important criteria for measuring students’ performance and giving them awards. … There were 30% of the total students who had been exempted from the course and their performance would be deemed as excellent by default because their score of English in the entrance examination was higher than 70. … According to the requirement that students’ performance of a class should be presented in a way of normal distribution, 70% students could by no means get an excellent score. … I didn’t know those 30% students, but I did feel some students of the 70% deserved an excellent score. I felt helpless, and struggled for some time, in facing the requirement from the institution and students’ actual performance.

*T8*: Some students who failed in the final exam only because of 2 or 3 points less than the passing score. I would feel nothing if I let the well-performed students pass, but it would be difficult for me to let the poorly-performed students pass. That was a kind of cheating and unfair to other students. … However, as a teacher I would also feel it a pity and even guilty for these poorly-performed students. … I was always in struggle while checking students’ exam papers.

The scoring system aims to evaluate students with formative rather than summative assessment. It is effective and comprehensive in evaluating students’ academic performance. However, since Chinese tertiary-level EFL teachers have the rights to change the final score, they are always faced with a dilemma. That is to say, they are not sure whether they should be strict enough to follow the rules or be tolerant enough to let students pass, especially those whose final score is 2 or 3 points less than the passing score. It is a matter of fairness, but it is also a matter of conscience.

### EFL Teachers’ Emotional Expressions

Unlike emotional experiences, EFL teachers talked little about their emotional expressions. This phenomenon is also reflected in the existing literature concerning studies on EFL teacher emotions. It might be due to the fact that the rollercoaster-like ups and downs of EFL teachers’ emotional experiences were instinctive and acute, which attracted their attention now and then, while their emotional expressions were constrained by the professional ethics which made their emotional expression undiversified.

*T6*: … As a teacher, as long as he/she enters the classroom, his/her emotional expressions are more or less affected by the profession. Restrain himself/herself. Control his/her emotions.

*T5*: Before entering the classroom, I will reflect on my emotional state at that moment. … If I find I am not in mood, I will adjust myself, trying not to be too emotional. …For example, if I undergo some bad things before the class, I will put them aside momentarily. I would not allow my class to be influenced by them. …

T6’s view was supported by almost all teacher participants. Especially, T5 explained this idea in great detail with her own experiences. These two excerpts show that these teachers were influenced by the traditional professional ethics and tended to display positive emotions by adhering to the laws, regulations, and social norms. For these teachers, the door shuts their negative emotions of daily life out of the classrooms where they were supposed to be professional. This is one of the reasons why EFL teachers’ emotional expressions are likely to be unanimous.

*T5*: At least, I will maintain an emotional state matching the lesson I am about to deliver. When I enter the classroom, I will set an emotion template or choose a relatively fixed emotion which depends on the lesson. If it is Intensive Reading which aims at imparting knowledge, authority is what I need so that students will follow my instruction. In this case, I would not display too many emotions. But if it is Oral English which requires more interaction with students, then I will show more warmth. … In such environment (classroom), I will try my best to control my emotion and am not likely to show too much personality. …

*T2*: It would not cause emotional fluctuation in me. Certainly, I was unhappy at that moment, but gradually I calmed down. … I told myself that I had to calm down and that I could accomplish the task successfully. I would call it a kind of psychological hint: I was prepared, and I could do it well.

*T6*: I was a little disappointed in this respect. I felt that if I was not good enough in language or communication skills, I would be disappointed at myself. … But I should be patient most of the time. No matter which emotion I was experiencing, I must control my emotions whenever I stood in the classroom as a teacher. Although I was very disappointed, I still had to smile, and explain slowly and patiently. …

*T7*: While facing such negative emotions as helplessness and depression, I could do nothing but tolerate.

It can be drawn from the above excerpts that these teachers were consistently as rational as they were supposed to be. T5 showed what most Chinese tertiary-level EFL teachers would usually do, a typical example of expressing teachers’ positive side to students. According to the division between surface acting and deep acting of emotional labor (Miller et al., 2007), T2, T5, and T6 were doing deep acting. They induced or suppressed their inner emotions to reflect the desired emotions. By contrast, T7 was doing surface acting. He suppressed his felt emotions by toleration. The difference between these two ways of displaying emotions lies in the fact that those who do deep acting tend to be initiative (see the report of T6 in the following excerpt) in teaching while those who do surface acting are likely to be inertial (see the report of T7 in the above excerpt).

*T4*: But I personally feel that I was more enthusiastic at the beginning when I started teaching in 2010, and then I had Buddhist-style gradually.

*T5*: (The non-English major students have to study English for) two years. My students and I tend to get along well in the first year, and in the second year, we would be fed up with each other. … (It was) aesthetic fatigue. … (I usually felt) annoyed. … As I became more familiar with their personality, I would express my preference or disgust easily. For example, I would like all students at the beginning. … But gradually I would get to dislike some of them when I learned more about their personality.

*T6*: When I felt disappointed, I couldn’t handle it properly, but I would make sure that the class would not be disturbed. After class, I would seek help from experienced teachers or read research papers concerning pedagogy so as to improve myself, and then apply the theories to my teaching practices…. I would acknowledge my limitation and realize that my students and I belong to different generations, which naturally hinders the communication between us. … Considering this reality, I would put myself in my students’ shoes and try to get to know things that they like. In this way, I would not feel so disappointed. …

As reported in the above excerpts, these teacher participants were anxious when they were novice teachers but also eager to display positive emotions. Even when they were experiencing negative emotions, they would induce the unfelt positive emotions or suppress the felt negative ones as had been required, especially when they taught a class of students for the first time. However, when they were gaining more experience in teaching or getting more acquainted with their students, they became indifferent to them, especially when students performed or behaved badly. In their terms, they were teaching in a Buddhist style, that is, they would take it as it came. What is worse, they were more likely to show negative emotions toward their students in the classroom after teaching for several years.

### Teacher-Student Interaction

The teacher-student interaction is chosen to observe the emotions of Chinese tertiary-level EFL teachers because it is one of the most important parts of EFL teachers’ teaching profession and the most direct source of teacher emotions. Normally, there are various ways for teachers to interact with students. The interactions can be in-class or out-of-class in formal or informal ways. After careful coding, extraction of codes, and classification of codes into themes, the interaction patterns of Chinese tertiary-level EFL teachers were found.

#### Physical Geography

EFL classroom is the major arena where EFL teaching takes place. The teacher participants unanimously reported that their interaction with students happened mainly in the classroom. Although these teachers admitted that interaction with students was necessary for and conducive to creating emotional climate in the EFL classroom, they regrettably admitted that they had few ways to interact with students.

*T1*: For example, I would ask (students) a question or a language point. “Did I explain the language point clearly? Is there any problem?” … or if the main task of the lesson I delivered was to do exercises, I had to ask them to answer the questions. … I seldom interacted with students after class. They had to do their homework, so I had to send them learning materials such as the slides I had used in the class.

*T10*: Most frequently, I interacted with students by asking questions. Of course, … as the class went on, students would ask questions too if they were puzzled. In this case, they were taking the initiative.

*T9*: I usually asked students to do presentation. Whenever the topic of a certain student’s presentation was interesting, the other students would also participate in the discussion, but generally it was me who made comments in the end.

It is obvious that these teachers seldom interacted with students. Asking questions seemed to be a preferable way that teachers would take. In this way, teachers, for example T9, were likely to follow a routine in teaching, making the class boring. As mentioned by T9, students’ interests were aroused not by his teaching, but by the contents that they were learning or their classmates’ way of doing presentation. Asking questions, therefore, would not work very well. Students would not buy it if teachers lent themselves to this way of interaction since “routine is a killer in the classroom” (Martínez Agudo, 2018, p. 6). What is worse, teachers could not guarantee that students were capable enough to answer all of the questions. It would usually lead to silence or even awkwardness in the classroom, which in turn adds to the emotional labor of teaching and affects teacher performance (Smith and King, 2018).

*T5*: In my eyes, work and life were separate, as a result of which my attitudes towards students were different. … There should be a golden section, from which you should not be too far away and to which you should not get too close either. Getting too close with students would exert bad influence on teaching. … Even though they were indifferent while responding to my questions, I found them nice. …I realized that I had had a cognitive bias towards them after the after-class chat. …

*T8*: … The after-class communication would increase students’ sense of identification with teachers.

Another problem is that these teacher participants reported that they seldom interacted with students after class, which is an important channel to get to know each other. Six in 10 teacher participants reported that they were reluctant to do so. Even when they did so that was only because they had to assign homework. They raised their concern that their work would mingle with their personal life. Therefore, they would like to keep some distance away from students after class. Such dilemma has caused a sense of helplessness among them. However, some teacher participants also mentioned that after-class interaction would be beneficial. For instance, T5 admitted that even though she was unwilling to interact with students after class, she had found her students’ bright side which was quite opposite to their performances in the classroom.

*T5*: The students were indifferent while I was asking questions. … After years of teaching, I was reluctant to ask them questions.

*T6*: In my first few years of teaching, I barely interacted with students. What I did was focusing on delivering language content knowledge, because as a novice I was too nervous to look at students.

T5 and T6 talked about the reasons for not interacting with their students. Their explanations were contradictory with each other. T5’s willingness to interact with her students was declining with the increase of her teaching experience because of her suffering of attrition. In contrast, T6 was too nervous to interact with his students due to his introvert personality. Teacher participants have also mentioned other factors hindering their interaction with students.

*T8*: I left school after class … and I could recognize only several students since there were too many students in my classes, about 40 to 50 students in a class, and I usually had 3 or 4 classes in one semester. … It took me a long time to know every one of the students. The fact was usually that I would not know all of my students before the semester was over.

*T6*: It's like this … My university was located in the outskirt, so I had to catch a shuttle bus traveling between campuses immediately after class. … I had classes only twice a week. … Students seldom had the chance to ask me questions if they did not take the chance of asking questions during the intervals.

In addition to teacher participants’ subjective factors as mentioned by T5 and T6, objective ones, such as time limitation and the number of students, and most probably learning materials and language content knowledge affected teacher-student interaction in one way or another.

#### Moral Geography

In this study, moral geography refers to the consistency between teachers’ and students’ pursuit of purposes and their sense of accomplishment (Hargreaves, 2001) in English learning. When teachers share the same purposes or have the same sense of accomplishment with students, they are in a moral geography of closeness; otherwise they are in a moral geography of distance.

*T1*: Then in this class, students often sat at the back of the classroom and talked a lot. Their voice was always louder than mine. In this case, I was irritated, and yes, it affected my feelings. … However, there was a sophomore in one of my classes who sat in the first row in every class, while the others sat in the last row. I really appreciated his attitude.

*T10*: While delivering a lesson in Class A, I could tell that students in Class A were joyful since they could understand what I was saying. It made me happy too. However, while I was teaching in Class B, students were not as joyful. They did not raise questions and their academic performances were not good enough. I felt sad sometimes and wondered how come they were so different.

In the above two excerpts, T1 and T10 made comparison between two different types of students. The comparison showed that moral agreement between teachers and students created a kind of closeness which led to the experience of positive emotions. T1 felt rewarded when that boy student’s action met her expectation that students should sit in the front of the classroom. T10 felt enjoyed when he taught in Class A because these students’ quick-witted acquirement of knowledge gave him a sense of accomplishment.

*T1*: … There is no sense of accomplishment. I have prepared the lesson carefully, and if I have not received a response, I feel that my efforts have not paid off.

*T2*: First, there would be a sense of boredom. There were always those few students who answered the questions, because I expected the other students to participate in the activities. …

*T6*: … I had a feeling of disappointment at the students because they did not respect me. I worked very hard to prepare the lessons. I have thought about all the possibilities, but the students did not respond, so I felt disappointed at the students and myself as well.

Hargreaves (2001) holds that teachers’ expectation to feel something good while teaching is culturally universal. EFL teachers are always expecting positive feedbacks and respects from students in classroom. Therefore, when they feel their purposes are threatened, their negative emotions will be triggered. T1, T2, and T6 did not get proper responses from students and felt their purposes failed, making them disappointed or bored. These emotions are detrimental since teachers experiencing them in teaching will gradually retreat inwards and lose energy and enthusiasm in EFL teaching.

*T6*: … I felt angry at that time. I thought I had done what a teacher should do, so I believed the topic I was dealing with was suitable for them. … They had no reason to ask me again or collect more test-related information from me. I regarded it as a distrust in me.

Among all the causes of moral distance, perhaps the worst one is students’ questioning over EFL teachers’ academic purposes, judgment, and expertise, which undermines Chinese tertiary-level EFL teachers’ confidence in their professionalism and makes them humiliated. For example, T6 was irritated by students’ repeated request for telling them the language points included in a test since it could be regarded as a sign of questioning his expertise in matching the difficulty of the test with students’ language proficiency.

#### Political Geography

Power or authority is a key element in teachers’ work. Borrowing the idea from Foucault and Janeway, Manke (1997) places the emphasis of teacher power on micro-interactional practice. It means that teachers enjoy power over students in the classroom. The traditional concept of teacher power believes that teachers have power because they have more professional knowledge than students. If students want to acquire knowledge, they must abide by school regulations and requirements and respect teachers’ power. In addition, as the possessor of knowledge, teachers play an authoritative role in the process of teaching and transferring knowledge. It can be seen that under the traditional view on teacher power, teachers have absolute control over students.

*T8*: … From my point of view, I hope to communicate with students equally, but as a matter of fact, teachers have and must have the dominant right or else the class might be in chaos since there are so many students in a classroom. So, I still hold the idea that teachers have to play a dominant role in classroom management.

In the view of T8, the power between teachers and students is necessary. As she said, keeping a distance away from students helps to establish teacher authority and manage classrooms. However, three participants, as is shown in the following excerpts, reported their powerlessness as a university teacher. The traditional view on teacher power has begun to change. Teacher power is no longer confined to teachers only but regarded as a bilateral power relationship between teachers and students. Both teachers and students share the power of controlling the learning environment. They construct power relations in the classroom together.

*T9*: I didn’t enjoy any power in the classroom.

*T5*: I didn’t have any authority. I was humble to the dust in front of my students.

*T2*: … On the one hand, the teaching position bestows authority on teachers; on the other hand, it is teachers’ knowledge structure that gives them authority. However, with the advancement of information and network technology, teacher authority is diminishing. … Students might have learned more from the Internet than teachers, which is a potential threat to teacher authority.

#### Sociocultural Geography

It is overtly obvious that teachers and students belong to different generations. The generation gap might cause a problem for teacher-student interaction.

*T1*: Students thought that teachers were teachers and students were students. They seldom share common topics.

*T6*: In order to know what my students were thinking, I had to listen to the songs and watched the TV series and movies they were interested in. Only in this way could I find the common ground with them. … While discussing the topic “knowledge is power”, one student argued that “money is power”. I was startled for a while and understood why this student would say that after I recalled that his parents were businessmen.

*T8*: If I wanted to discuss with my students, I usually chose topics close to their life.

Moreover, teachers and students grew up in different environments. Students’ family and the social-economic environment were the most frequently mentioned factors that influenced teacher-student interaction. Students had shaped a completely different outlook from that of their teachers. Even so, the teachers would still try to cater for their students’ interests by talking or behaving in their ways.

*T3*: Teachers should be careful in wording since these students did not like teachers who were serious. Teachers should communicate with them in a negotiating way.

*T5*: The post-90s generation have grown up in a wealthier family, which made them sensitive to emotions and more ego-centered and cared more about themselves.

*T10*: These post-90s students were active in thinking even though they were sensitive to criticism. … I found that those who were from wealthy families were good at interacting with me. Instead, those who were from poor families were too shy to raise a question.

These three excerpts show that environment is one of the determinants that shape students’ personality and emotions. The influence could be either positive or negative. On the one hand, what surprised the three teacher participants most was the fact that students who grew up in these environments were ego-centered and were more sensitive to emotions. On the other hand, these students enjoyed some advantages in establishing interpersonal relationship as T10 mentioned.

#### Professional Geography

Chinese EFL teachers were usually told to be kind to students when they were still teacher students and required to love students when they attended the in-service professional training. Chances are, therefore, that Chinese EFL teachers usually show their kindness, love, and care to their students at the beginning of their teaching professions.

*T4*: The school required teachers to care about students.

T1/T7: It is the duty and responsibility of teachers to love every student.

Teacher participants reported that as they and their students got to know each other, their students tended to be intimate with them. It worried them a lot. As far as these teacher participants were concerned, keeping away from students was maintaining the teachers’ authority. The intimacy of relationship between teachers and students undermined their authority and eventually spoiled teaching in the classroom. This attitude is extremely supported by T5 who suggested that there should be a golden section between teachers and students.

## Discussion and Conclusion

This paper reports on interviews with 10 Chinese tertiary-level EFL teachers in terms of their emotional experiences, emotional expressions, and their interactions with students. It aimed at drawing a clear picture of these EFL teachers’ emotional lives at school and sought to unearth underlying factors influencing their emotional experiences, expressions, and interactions with students.

### Emotional Experience and Expression

In the present study, the positive emotions most frequently reported by teacher participants were enjoyment and pride, a result providing more supplementary empirical evidence to previous studies (Sutton and Wheatley, 2003; Frenzel et al., 2016). Teacher participants reported positive emotions mainly because of the fact that students followed the classroom rules, that students’ performance exceeded teachers’ expectation, or that students gained outstanding success in competitions. These roots of positive emotions are also discussed in Xu’s (2013) study which suggests that the emotional rewards of teaching are mostly from students. The qualitative study also shows an interesting result that a small proportion of EFL teachers felt excited because of the vigor and energy that their young students brought to the classroom. Some teachers even regarded it as a source of encouragement in their daily work by saying that they loved working with teenagers and that whenever they saw these students full of energy and imagination they found themselves younger too.

Unlike their counterparts in primary schools, Chinese tertiary-level EFL teachers weigh more on individual students’ unexpected performance than on the affection from students. Positive emotions experienced by elementary teachers are rooted in the teacher-student interaction in the classroom (Hargreaves, 2000). In order to gain the emotional rewards, elementary teachers usually try to work emotionally in order to be more effective in their teaching (Woods and Jeffrey, 1996). They go out of their way to establish emotional bonds with students by generating positive emotions, such as excitement, enjoyment, and enthusiasm, be it genuine or faked. Moreover, elementary teachers speak highly of being missed by their students; being their students’ favorite teacher; or being loved by their students. Compared with their counterparts in secondary schools who placed much value on students’ respect, only one in 10 participants ascribed their positive emotions to the respect from students. This might be because university students are mature enough not to show disrespect in front of their teachers nor to mock their teachers, making student respect a less important antecedent of Chinese tertiary-level EFL teachers’ emotions. Similarities, however, lie in the fact that both secondary school teachers in Hargreaves’ (2000) study and tertiary-level EFL teachers in the present study admitted that positive emotions come from achieving breakthroughs with individual students.

Negative emotions are an inseparable part of teacher emotions. It is true at least in the present study where teacher participants reported more negative emotions than positive ones. Many would attribute this to such cause as the participants’ emotional burnout and attrition after several years of EFL teaching. It makes sense because most of our participants had a teaching experience ranging from 6 to 10years and they were approaching their 40s, where there exists the highest level of emotional exhaustion according to a meta-analysis of university teachers’ professional burnout (Zhou et al., 2019). However, this finding does not support the convention that teachers are supposed to be positive in emotion and to show warmth to students in an eastern culture like that of Chinese and Japanese. For example, experienced Japanese tertiary-level EFL teachers have very positive emotions toward their students by acting as careers and moral guides while interacting with their students (Cowie, 2011). Although these teachers experienced negative emotions frequently, they managed to hide them and pretended to be positive most of the time. One of the participants stated that she would separate work from life and would not allow her teaching to be affected by her personal out-of-classroom emotions. As a result, she would adjust herself and wear a smiling face whenever she entered the classroom.

Apparently, there is a disjunction between emotions they experienced and those they expressed. The factor drawing the division is the emotional labor or emotional rule that Benesch (2012, 2017) proposes. The purposeful expression of positive emotions and suppression of negative emotions are typical examples of emotional labor in school settings. When EFL teachers in our study experienced such negative ones as anger, anxiety, and discouragement in the EFL classroom, they suppressed them by giving up the current teaching task, stopping the conflict with students or asking students to learn by themselves, etc. What they did was to express personal emotional effort, emotional planning, and emotional restraint in accordance with the emotional rules of the school context (Morris and Feldman, 1996).

Apart from the root of negative emotions in emotional exhaustion and emotional labor, EFL teachers’ experience of negative emotions might also be caused by bad relationships or no relationships (Oatley, 1991). Opposite to elementary teachers who are constantly in emotional intensity with their students, university EFL teachers and secondary teachers are troubled by emotional misunderstanding and emotional distance in their classrooms. It is exceedingly difficult to make emotional understanding and connection with large numbers of students. In China, the English course for non-English major students has been squeezed to less than 4 or even fewer class hours per week and the classrooms have been crowded with at least 40 students. The number might even be as high as 80 in a single classroom. As a result, on the one hand, EFL teachers are in a rush to finish the content knowledge in a limited time schedule; on the other hand, even if EFL teachers intend to interact with students, the excessive number of students makes it difficult for them to remember each student’s name in a short period of time, let alone interact with them effectively.

Special attention should be paid to ambivalence reported by the participants since it is seldom discussed in the existing literature. Even though some discrete emotions are not salient in education, their influences in relation to teaching practice are worth discussing, as Martínez Agudo and Azzaro (2018) rightly point out. The participants reported that they were torn between students’ request for giving a passing score and the institution’s rules of keeping the scores of all students in normal distribution and the intention to treat each student with equity and fairness. In other words, these teachers’ empathy and concern for students conflicted with the institution’s rules and their professional ethics. In spite of the fact that such emotion is not detrimental, it undermines EFL teachers’ enthusiasm in teaching.

### Teacher-Student Interaction and Its Role as Antecedents of Teacher Emotions

Teacher-student interaction is regarded as the momentum pushing the teaching forward. The effectiveness of teacher-student interaction can be observed by the emotional closeness and distance between teachers and students. According to the emotional geography theory, the pattern of teacher-student interaction in Chinese tertiary-level EFL class is drawn.

First of all, the teacher-student interaction at tertiary-level features the emotional geography of physical distance. The closeness and/or distance created by time and space becomes the basic configuration in the EFL classroom of Chinese universities because their interaction is usually segmented. Similarly, secondary school teachers keep a physical distance away from students because of the well-elaborated timetable for subjects that divides students among many teachers and the overloaded burden of cognitive content (Hargreaves, 2000). These prominent factors, among many others, fragment the interactions between teachers and their students, making emotional understanding difficult to achieve. The time for interaction is limited to once or twice a week and the space is also confined in the EFL classroom at tertiary-level. Considering the limitations in time and space, EFL teachers prefer formal, in-class interaction with their students and they ubiquitously interact with students by way of the Initiate, Response, and Evaluation (IRE) model (Zemel and Koschmann, 2011). The common practice in EFL classroom follows the IRE sequence, that is, the teacher initiates the interaction with a question, students respond to it, and then, the teacher gives evaluation on students’ responses.

In the interview, eight of our participants interacted with students in the IRE way. They did this because they gave priority to the realization of teaching goals of each individual lesson. Therefore, the class usually went on according to the scripts made in advance. Our participants admittedly had to resort to this approach to interact with their students who did not engage in the classroom activities. However, students who were passive in the EFL classroom interacted with teachers actively after class. Two participants reported that the informal interaction with students after class changed their impression of them. University EFL teachers usually found their students energetic, warm-hearted, and active after class, which was similar to Hargreaves’ (2000) observation of secondary teachers. This informal and unexpected after-class interaction serves as a source of EFL teachers’ positive emotion. Undoubtedly, this may also have a reciprocal effect on students, helping them see their teachers in a new light.

Second, moral distance is also a prominent feature of interactions between tertiary-level EFL teachers and their students. Moral distance may refer to the divergence of what teachers believe from what students believe. Such divergence is one of the major threats of EFL teacher enjoyment. Xu (2013) finds that EFL teacher happiness depends on the achievement of their educational/teaching purposes. This explains why EFL teachers try every means to ensure that their teaching goals are realized at the end of each lesson. If this purpose is disrupted by students’ misbehavior or other unexpected things, they will feel upset; but if students’ performances meet their requirement, they will be happy. For example, T10 felt offended when his student defied him by questioning him about the usefulness of learning English. Chinese students are expected to obey what they are asked to do (Xu, 2013). T1 was moved by one boy student when she saw him sit in the first row while the others were crowded at the back. Most importantly, our results also echo with Hargreaves’ (2001) finding that questioning EFL teachers’ academic purposes and expertise was the direct source of such acute negative emotions as anger.

Third, political distance is common in schooling since teachers are endowed with power by the nature of teaching profession. Teacher participants in the present study wanted to keep a political distance away from their students. It can be explained by their intention that they hoped to make their power explicit by keeping a distance. In this way, they seemed to have the legitimate right to order students to follow their instructions obediently. It was their selfish desire to maintain their classrooms in order that was at work. They wished to have absolute authority which primary and secondary teachers enjoyed, making them reluctant to change their role as a university teacher. What the qualitative results show us, however, is a different picture. Seven teacher participants reported that they were losing teacher power. Three teacher participants complained that they did not have the power at all. As a matter of fact, student behavior is negotiated between university teachers and students. This is why university students try to request their teachers to change the score of final exams or to offer the contents to be covered in the testing paper, which will never happen in the primary and secondary schools. When students do so, university EFL teachers may feel their power is challenged, which generates EFL teachers’ negative emotions.

This finding suggests that Chinese tertiary-level EFL teachers are politically close to students, which is quite opposite to Xu’s (2013) observation. The reason can be that her study was carried out in middle schools where EFL teachers possessed more classroom power than university teachers because of the differences between teachers and students in age, physical size, and strategic sophistication. When it comes to university schooling, students become adults whose mentality is mature enough to think of sophisticated strategies independently. In this sense, university students are no longer in a relationship of submission to their teachers. In addition, in this information age, the information and knowledge gaps between teachers and students are much narrowed. Teachers are no longer the only source for students to acquire knowledge. These two changes contribute to the diminishment of teacher power in the EFL class at tertiary-level.

To sum up, the present study reveals that Chinese tertiary-level EFL teachers experience more negative emotions than positive ones, but, more often than not, they have to suppress negative emotions, which is required by the emotional rules in school settings. It may lead to emotional labor which causes more negative emotions. As for the patterns of teacher-student interaction in Chinese tertiary-level EFL classroom, the configuration features physical and moral distances, and political closeness, which are potential forces leading to emotional misunderstanding and in relation to EFL teachers’ negative emotions.

## Suggestions and Limitations

This study and those documented in the literature clearly show that language teaching incorporates factors causing a series of negative emotions. Generally, teachers are recommended to take realistic-positive attitudes to cope with negative emotions (Wilson, 2002), which turns out to cause more frustration. In view of the predominance of negative emotions experienced by Chinese tertiary-level EFL teachers, we contend that interventions from the positive psychology perspective are necessary in order to foster teachers’ wellbeing and positivity (Meyers et al., 2013), improve their mental health and productivity, and enhance the quality of teaching and learning (Rahm and Heise, 2019). For example, Jin et al. (2021) find that people with dispositional optimism and experiencing more positive emotions are likely to experience higher wellbeing and better resilience, suggesting that teaching educators’ strategies of gratitude, positive outlooks, and optimism help them develop psychological resources to cope with stressors. Gregersen et al. (2020) conduct a Silver Linings intervention asking their participant to pay attention to problems arising from her classes or her life outside of school and search for silver linings in a difficult context. Still, however, positive psychological interventions (PPIs) have seldom been applied to alleviate EFL teachers’ stress and decrease their experiences of negative emotions.

Positive Psychological Interventions are defined as “intentional activities aimed at cultivating positive feelings, positive behaviors, or positive cognition” (Sin and Lyubomirsky, 2009: 467) on the basis of central theoretical concepts of positive psychology, such as gratitude, strengths, optimism, and kindness. PPIs in foreign language teaching include any intentional activities or methods tailored to a specific context of applied linguistics or TESOL to facilitate growth and development among language learners (Gregersen et al., 2016), and, we believe, among EFL teachers as well. Therefore, we agree with Meyers et al. (2013) who propose that PPIs be carried out in three ways: “(a) the cultivation of positive subjective experiences, (b) the building of positive individual traits, or (c) the building of civic virtue and positive institutions” (p. 620). Accordingly, teacher educators should first provide intervention programs aiming at helping EFL teachers understand temporal and subjective positive experiences, such as to recall moments that make teachers grateful or experience positive emotions, or to look in a positive way at the difficult situations. Then, teacher educators should provide intervention programs aiming at identifying, developing, and/or broadening valued permanent and stable individual traits or trait-like constructs (e.g., character strengths). Finally, PPIs regulate not only individual EFL teachers’ mindset, but also the characteristics of schools. It means that teacher educators and researchers, even policy-makers, should also identify, develop, broaden, and/or put to practice the valued characteristics of organizations while carrying out intervention programs directing at individual EFL teachers.

This study has unfolded a panorama of Chinese tertiary-level EFL teachers’ emotional lives, which is helpful for researchers to understand the emotional efforts that EFL teachers invest in the emotion-charged EFL teaching. However, a major limitation is the small number of participants. The results derived from the 10 interviews can only account for a small part of the reality of EFL teachers’ emotional lives, making the study subject to criticism. Moreover, EFL teachers’ emotional expression, one of the major aims of the study, was not reported as frequently as their emotional experience and its effects on EFL teaching were not discussed thoroughly. Finally, although possible ways of reducing EFL teachers’ experiences of negative emotions have been proposed, devising applicable PPI programs in Chinese context still remains to be done in future studies.

## Data Availability Statement

The raw data supporting the conclusions of this article will be made available by the authors, without undue reservation.

## Ethics Statement

The studies involving human participants were reviewed and approved by the College of Foreign Languages and Cultures, Xiamen University. The patients/participants provided their written informed consent to participate in this study.

## Author Contributions

XX and GJ contributed to conception and design of the study, and performed the data analysis. XX conducted the interviews and transcribed them verbatim, and wrote the first draft of the manuscript. Both authors contributed to manuscript revision, read, and approved the submitted version.

## Funding

The present article is part of the research project “Non–verbal emotional interaction and the effectiveness of foreign language classroom teaching,” supported by the National Social Science Foundation of China (Grant No: 15BYY082).

## Conflict of Interest

The authors declare that the research was conducted in the absence of any commercial or financial relationships that could be construed as a potential conflict of interest.

## Publisher’s Note

All claims expressed in this article are solely those of the authors and do not necessarily represent those of their affiliated organizations, or those of the publisher, the editors and the reviewers. Any product that may be evaluated in this article, or claim that may be made by its manufacturer, is not guaranteed or endorsed by the publisher.
